# T1R3 homomeric sweet taste receptor regulates adipogenesis through Gαs-mediated microtubules disassembly and Rho activation in 3T3-L1 cells

**DOI:** 10.1371/journal.pone.0176841

**Published:** 2017-05-04

**Authors:** Yosuke Masubuchi, Yuko Nakagawa, Johan Medina, Masahiro Nagasawa, Itaru Kojima, Mark M. Rasenick, Takeshi Inagaki, Hiroshi Shibata

**Affiliations:** 1 Department of Molecular and Cellular Biology, Institute for Molecular and Cellular Regulation, Gunma University, 3-39-15 Showa-machi, Maebashi, Japan; 2 Departments of Physiology & Biophysics and Psychiatry, University of Illinois, Chicago, Illinois, United States of America; and the Jesse Brown VA Medical Center, Chicago, Illinois, United States of America; Duke University, UNITED STATES

## Abstract

We previously reported that 3T3-L1 cells express a functional sweet taste receptor possibly as a T1R3 homomer that is coupled to Gs and negatively regulates adipogenesis by a Gαs-mediated but cAMP-independent mechanism. Here, we show that stimulation of this receptor with sucralose or saccharin induced disassembly of the microtubules in 3T3-L1 preadipocytes, which was attenuated by overexpression of the dominant-negative mutant of Gαs (Gαs-G226A). In contrast, overexpression of the constitutively active mutant of Gαs (Gαs-Q227L) as well as treatment with cholera toxin or isoproterenol but not with forskolin caused disassembly of the microtubules. Sweetener-induced microtubule disassembly was accompanied by activation of RhoA and Rho-associated kinase (ROCK). This was attenuated with by knockdown of GEF-H1, a microtubule-localized guanine nucleotide exchange factor for Rho GTPase. Furthermore, overexpression of the dominant-negative mutant of RhoA (RhoA-T19N) blocked sweetener-induced dephosphorylation of Akt and repression of PPARγ and C/EBPα in the early phase of adipogenic differentiation. These results suggest that the T1R3 homomeric sweet taste receptor negatively regulates adipogenesis through Gαs-mediated microtubule disassembly and consequent activation of the Rho/ROCK pathway.

## Introduction

The sweet taste receptor in the taste buds is a heterodimer composed of two class C G protein-coupled receptors (GPCRs), T1R2 and T1R3 [1]. This T1R2/T1R3 heterodimer is expressed in type II taste cells in the taste buds and is activated with various sweet compounds such as sugars, artificial sweeteners, sweet amino acids, sweet proteins and stevioside [2]. A widely-accepted signaling mechanism downstream of the sweet taste receptor is that the T1R2/T1R3 heterodimer couples to α-gustducin (Ggust) or other yet undefined G protein(s), causing activation of phospholipase Cβ and subsequent calcium release. This, in turn, elicits sodium ion influx through TRPM5 channel and membrane depolarization, leading to the release of ATP, the transmitter of type II taste cells [3]. Over the past years, an increasing number of reports have demonstrated that taste receptors are also expressed in a variety of nongustatory cell types, suggesting that those taste receptors may have additional physiological functions (for review see [4]). In this context, we previously reported [5] that T1R3 was expressed in 3T3-L1 preadipocytes as well as in primary adipose tissue-derived stromal cells and its expression was markedly up-regulated during adipogenic differentiation, while the expression of T1R1 and T1R2 remained at a very low level. Such disproportionate expression profiles of T1Rs suggested that, unlike the case in taste cells, T1R3 might be expressed as a homomeric form in adipocytes. Stimulation of this putative T1R3 homomeric receptor significantly attenuated adipogenesis by a Gαs-mediated but cAMP-independent mechanism [5] although its precise downstream signal is yet to be defined.

On the other hand, the physiological significance of sweet taste receptor(s) in adipogenesis and energy metabolism has not been fully clarified. Thus, in contrast to our observations, Simon et al. reported that saccharin and acesulfame potassium stimulated adipogenesis in mouse mesenchymal stem cells and human preadipocytes although the effects were independent of T1R2 and T1R3. The same group has shown that both T1R2 and T1R3 knockout mice have reduced adiposity and smaller adipocytes on a Western Diet. Additionally, those T1R3 deficient mice were with mild glucose intolerance and no changes in insulin sensitivity, while Murovets et al. reported that T1R3 knockout mice on a standard diet showed substantially reduced glucose tolerance and insulin sensitivity. These studies suggest that sweet taste receptor, especially T1R3, is possibly involved in the control of glucose metabolism although the reasons for the inconsistent results remain obscure.

In the present study, we investigated the Gαs-dependent but cAMP-independent anti-adipogenic signals downstream of the T1R3 homomeric sweet taste receptor in 3T3-L1 cells. Among recently reported non-canonical roles of heterotrimeric G proteins (for review see [6]), Gαs-mediated tubulin GTPase activation and microtubule disassembly are one possible mechanism for the T1R3 homomeric receptor-mediated inhibition of adipogenesis, since microtubule disassembly causes the release and activation of GEF-H1 (ARHGEF2), a microtubule-bound RhoGEF [7], which activates the Rho GTPase, a negative regulator of adipogenesis [8–11]. Here we tested this working hypothesis. The results of the present study provide evidence that the T1R3 homomeric sweet taste receptor regulates adipogenesis through Gαs-mediated microtubules disassembly and Rho activation in 3T3-L1 cells.

## Materials and methods

### Materials

Rabbit antibodies for GEF-H1, myosin phosphatase targeting protein 1 (MYPT1), phospho-MYPT1 (Thr696), Akt, phospho-Akt (Ser473), FoxO1, phospho-FoxO1, PPARγ, and C/EBPα were obtained from Cell Signaling Technology, Inc. (Danvers, MA). Rabbit polyclonal anti-GEF-H1 antibody was also purchased from Abcam (Cambridge, UK) and used for immunostaining. Mouse monoclonal anti-tubulin (clone TUB 2.1) and anti-actin (clone AC-40) antibodies and sucralose were obtained from Sigma (St. Louis, MO). Sodium saccharin, cholera toxin and Y-27632 were from Wako Pure Chemical Industries (Osaka, Japan).

### Cell culture and differentiation

3T3-L1 cells provided by Howard Green (Harvard Medical School, Boston, MA) [12] were maintained in Dulbecco’s modified Eagle’s medium containing 4.5 g/L D-glucose (DMEM-HG) supplemented with 50 μg/ml penicillin, 75 μg/ml streptomycin and 10% calf serum (CS) at 37°C in a humidified atmosphere of 5% CO_2_, and were differentiated into adipocytes as described previously [13]. Briefly, 2 days after confluence, the medium was replaced with fresh DMEM containing 1.0 g/L D-glucose (DMEM-LG) supplemented with 10% fetal bovine serum (FBS), 0.5 mM 1-methyl-3-isobutylxanthine (IBMX), 10 μM dexamethasone, and 1.7 μM insulin. Forty-eight hours later, the medium was replaced with fresh DMEM-LG containing 10% FBS and 1.7 μM insulin. After 48 hours, insulin was withdrawn from the culture media and cells were maintained in DMEM-LG containing 10% FBS.

### Immunoblotting

For immunoblot analysis of GEF-H1, MYPT, phospho-MYPT, PPARγ, C/EBPα, cells were washed with PBS, lysed in Laemmli buffer, boiled and centrifuged for 10 min at 10,000 rpm at 4°C. The supernatant was subjected to SDS-PAGE and immunoblotting. For immunodetection of T1R3, cells were homogenized in PBS containing Complete protease inhibitor cocktail (Roche) and PhosSTOP phosphatase inhibitor cocktail (Roche), followed by centrifugation for 5 minutes at 7,500 rpm at 4°C. The supernatant was subjected to SDS-PAGE and immunoblotting. The blots were visualized by using Amersham ECL detection systems (GE Healthcare) and LAS-4000 luminescent image analyzer (GE Healthcare). The intensities of the bands were quantified by using Multi Gauge software (Fuji Photo Film, Tokyo). The protein amount was normalized with the amount of β-tubulin or actin as internal controls by either reprobing the each PVDF membrane or immunoblotting the same sample with anti-β-tubulin or anti-actin antibodies.

### Immunostaining

3T3-L1 cells grown on a cover slip were fixed with 3% (w/v) paraformaldehyde for 10 minutes at room temperature or with 100% methanol for 2 minutes at -20°C, and immunostained with anti-β-tubulin and anti-GEF-H1 primary antibodies and Alexa Fluor 488- or Alexa Fluor 568-conjugated secondary antibodies as described previously [13]. The actin filaments were stained with Alexa Fluor 568-conjugated phalloidin (Thermo Fisher Scientific). Cells were also stained with 4',6-diamidino-2-phenylindole (DAPI) to visualize the nuclei. Immunofluorescence images were obtained with FluoView FV1000 confocal microscope system (Olympus, Tokyo).

### Oil Red-O staining

Differentiated 3T3-L1 cells at Day 6 were washed twice with PBS and fixed in 3% (w/v) paraformaldehyde in PBS for 10 min at room temperature. After washing twice with PBS, cells were incubated with 60% isopropanol solution for 1 minute before staining with Oil Red-O solution (3 mg/mL 60% (v/v) isopropanol) for 20 min. Cells were washed once with 60% (v/v) isopropanol and twice with PBS before observation by microscopy. For quantification of the amount of Oil Red-O, the dye was extracted by incubation of the cells with 100% isopropanol for 20 min, and the absorbance at 518 nm was measured.

### Transfection of plasmid DNA or siRNA

The cDNA for wild-type rat Gαs was provided by Randall R. Reed (Johns Hopkins University, Baltimore, MD) and subcloned into the pCMV5 expression vector [14]. The cDNA constructs for Gαs-G226A or Gαs-Q227L were prepared by using QuikChange II site-directed mutagenesis kit (Agilent Technologies). The human RhoA expression plasmids, pEF-BOS-Myc-RhoA and pEF-BOS-Myc-RhoA-T19N, were kindly provided by Takashi Matozaki (Kobe University). The expression vector for Gαs-GFP fusion protein with green fluorescent protein (GFP) inserted into an internal loop of Gαs was described before [15]. Small interfering RNA (siRNA) duplexes targeting for mouse GEF-H1 (Table 1) were purchased as Dharmacon siGENOME SMARTpool from Thermo Fisher Scientific Inc. (Waltham, MA).

**Table 1:** Target sequences for siRNA.

Target	Gene symbol	Target sequence of mRNA
GEF-H1	Arhgef2	GUACCAAGGUCAAGCAGAA (257–275) CAACAUUGCUGGACAUUUC (459–477) UGGAAUCCCUUAUUGAUGA (506–578) GCACUGGGAUGCUGGAAGA (788–806)

3T3-L1 preadipocytes grown on a culture dish were dispersed with 0.05% trypsin in PBS. After washing three times with PBS, cells were resuspended in Electroporation Buffer (Bio-Rad). A cell aliquot was mixed with the expression plasmid (20–30 μg) or with the siRNA duplexes (5 nmole) in a 0.4 cm-gap cuvette before electroporation by using Gene Pulser Xcell (Bio-Rad) set at 200 V and 28 msec in a time-constant mode. Electroporated cells were resuspended in DMEM-HG containing 10% CS and seeded on a culture dish.

### Rho activity assay

The activity of the Rho GTPase was measured by pull-down assay using the Active Rho Detection Kit (Cell Signaling Technology, Inc.) according to the manufacturer’s instructions.

### Time-lapse imaging of Gαs-GFP

3T3-L1 preadipocytes were transfected with 30 μg of the Gαs-GFP expression plasmid by electroporation and seeded on a 35 mm glass bottom culture dish. After incubation for 24 hours, medium was removed and replaced with Hanks’ balanced salt solution (HBSS) containing 138 mM NaCl, 5.4 mM KCl, 1.3 mM CaCl_2_, 0.5 mM MgCl_2_, 0.38 mM MgSO_4_, 0.44 mM KH_2_PO_4_, 0.34 mM Na_2_HPO_4_, 5.5 mM D-glucose and 20 mM Hepes/NaOH, pH 7.4 before stimulation. Cells were excited with the wavelength of 488 nm and the fluorescence images of Gαs-GFP were captured at 15-second intervals with a 12-bit C7780-22 ORCA3CCD camera (Hamamatsu Photonics, Hamamatsu, Japan). The fluorescence intensity (F) from each ROI (region of interest) was normalized to the initial value (F_0_) so that the relative fluorescence change was referred to as F/F_0_.

### Real-rime measurement of Rho activity

The cellular activity of RhoA GTPase was measured by using a FRET (fluorescence resonance energy transfer)-based Rho probe, Raichu1237X [16] provided by Dr. Michiyuki Matsuda (Osaka University). RaichuX1237 consists of truncated RhoA (aa 1–189) and the RhoA-binding domain (RBD) of protein kinase N (PKN) (aa 13–98) fused between cyan fluorescent protein (CFP) and yellow fluorescent protein (YFP). The intramolecular binding of GTP-RhoA to the RBD induces a conformational change leading to a decrease in the distance between the fluorophores, which is measured as an increase in FRET from CFP to YFP. Briefly, cells were transfected with 30 μg of plasmid encoding Raichu1237X by electroporation and seeded on a 35 mm glass bottom dish. After incubation for 24 hours in DMEM-HG with 10% CS, medium was removed and replaced with HBSS. For measurement of RhoA activity, cells were excited with 440 nm light and dual emission images of CFP and YFP were obtained using the AQUACOSMOS/ASHURA fluorescence resonance energy transfer imaging system (Hamamatsu Photonics, Hamamatsu, Japan). The data are presented as the emission ratio of YFP/CFP.

### Statistical analysis

Data were analyzed by Student’s t-test and P < 0.05 was considered as statistically significant.

## Results

**Fig 1:**
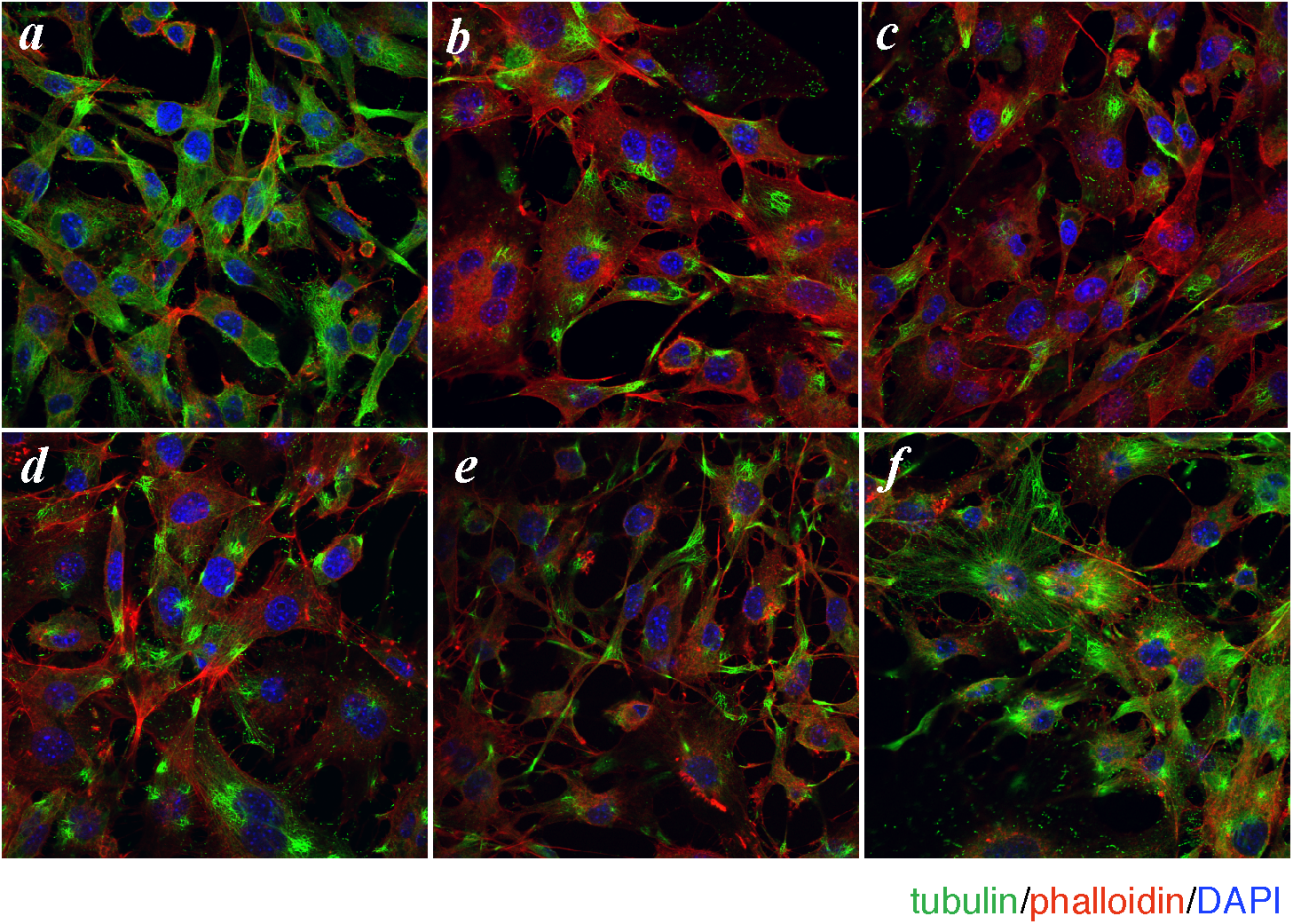
Sweeteners and Gαs-activating agents cause microtubule disassembly in 3T3-L1 cells. 3T3-L1 cells grown on a cover glass were treated without (control) (*a*) or with sucralose (20 mM) (*b*), saccharin-Na (20 mM) (*c*), isoproterenol (5 μM) (*d*), cholera toxin (0.1 μg/ml) (*e*) or forskolin (40 μM) (*f*) in DMEM-LG for 2 hours (except with cholera toxin 6 hours). Cells were then fixed and immunostained for β-tubulin (green). Filamentous actin (red) and cell nuclei (blue) were visualized with Alexa Fluor 568 phalloidin and DAPI, respectively.

To test our working hypothesis that the anti-adipogenic signal of the T1R3 homomeric sweet taste receptor would be transmitted through Gαs-induced microtubule disassembly and subsequent activation of Rho GTPase, we first examined the effects of sucralose or saccharin on microtubule integrity in 3T3-L1 preadipocytes. As shown in Fig 1, the addition of sucralose or saccharin for 2 hours caused marked disruption of the microtubule network in 3T3-L1 preadipocytes (Fig 1B and 1C). Two other Gαs activators, isoproterenol and cholera toxin, but not the adenylyl cyclase activator, forskolin, also evoked disassembly of microtubules (Fig 1D–1F). These results suggested that Gαs activation but not cAMP generation is involved in sweetener-induced microtubule disassembly. Notably, the phalloidin signals were increased in the microtubule-disrupted cells (Fig 1B and 1C), suggesting that Gαs may regulate both microtubules and actin cytoskeletons.

**Fig 2:**
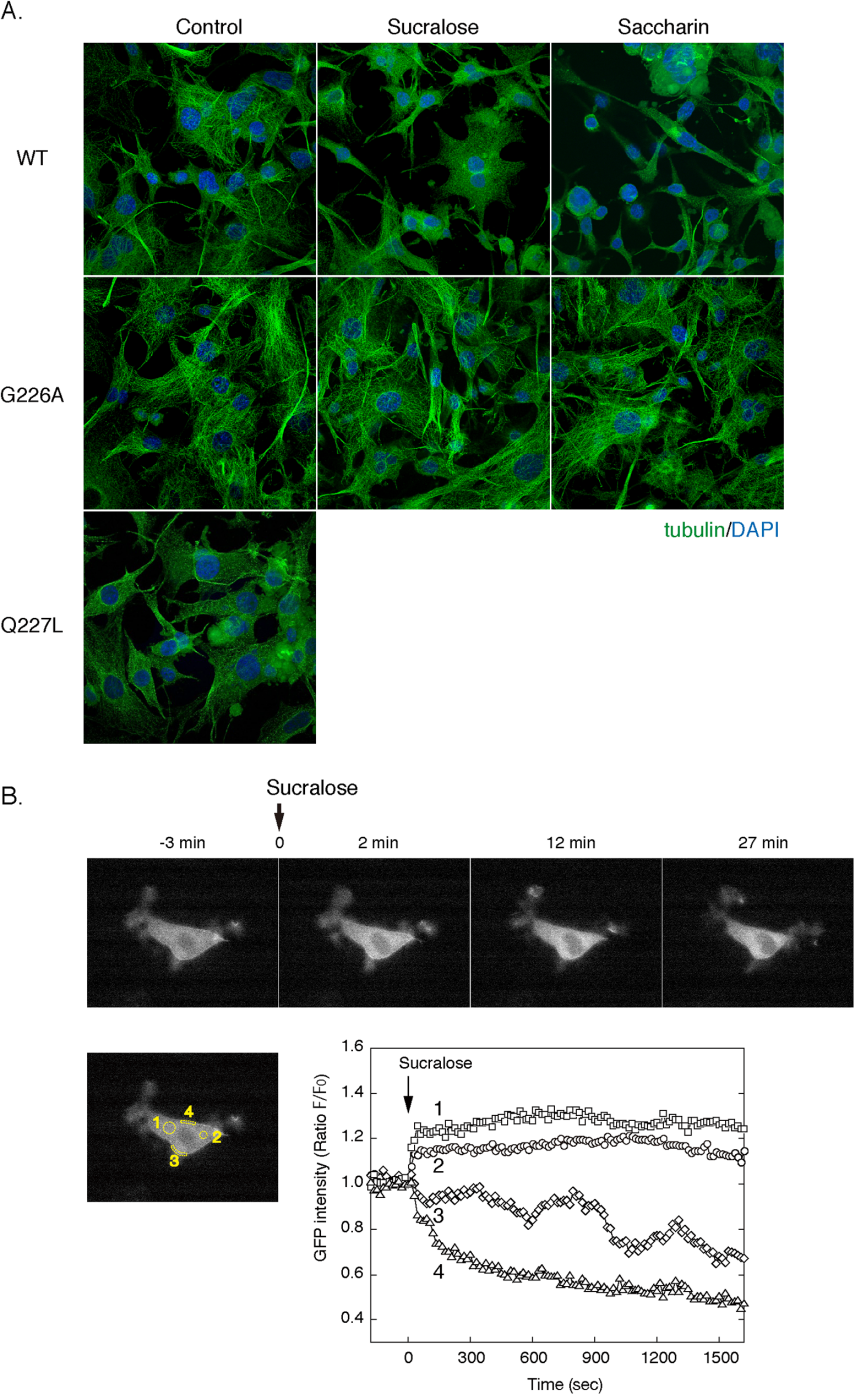
Role of Gαs in microtubule disassembly with sweeteners. A. 3T3-L1 cells were transfected by electroporation with the expression plasmids (30 μg each) for Gαs (wild-type), Gαs-G226A or Gαs-Q227L and were cultured for 24 hours. Cells were then treated without (control) or with sucralose (20 mM) or saccharin (20 mM) for 3 hours in DMEM-LG. Cells were fixed and immunostained for β-tubulin (green). Cell nuclei (blue) were visualized with DAPI. B. 3T3-L1 cells transfected with the expression plasmid for Gαs-GFP (30 μg) were stimulated with 20 mM of sucralose at the indicated time point. The GFP fluorescence images were obtained at 15 sec intervals. The images at the indicated time points were shown in the upper panel. The fluorescence intensities from the cytoplasm (ROIs 1 and 2) as well as from the cell periphery (ROIs 3 and 4) were shown in the lower panel.

To further investigate the role of Gαs in the regulation of the microtubules, we overexpressed dominant-negative (Gαs-G226A) and constitutively active (Gαs-Q227L) mutants of Gαs. As shown in Fig 2A, overexpression of Gαs-G226A blocked the effects of sucralose and saccharin on the microtubules. In contrast, overexpression of Gαs-Q227L induced disassembly of the microtubules in the absence of sweetener stimulation (Fig 2A). These results support the notion that stimulation of the T1R3 homomeric sweet taste receptor causes microtubules disassembly by a Gαs-dependent mechanism. Previous studies have demonstrated that GTP-bound Gαs internalizes and destabilizes microtubules by directly stimulating the tubulin GTPase[17, 18]. We thus examined, via real-time imaging using the Gαs-GFP fusion protein, whether stimulation of the T1R3 homomeric sweet taste receptor would evoke a subcellular shift of Gαs. As shown in Fig 2B, Gαs-GFP localized to the cell periphery of 3T3-L1 preadipocytes before sucralose stimulation. Sucralose stimulation caused a rapid increase in intracellular fluorescence (ROI 1 and 2), which was accompanied by a reciprocal decrease in the GFP signal on the plasma membrane (ROI 3 and 4), suggesting a subcellular shift of Gαs-GFP from the plasma membrane to the cytoplasm.

**Fig 3:**
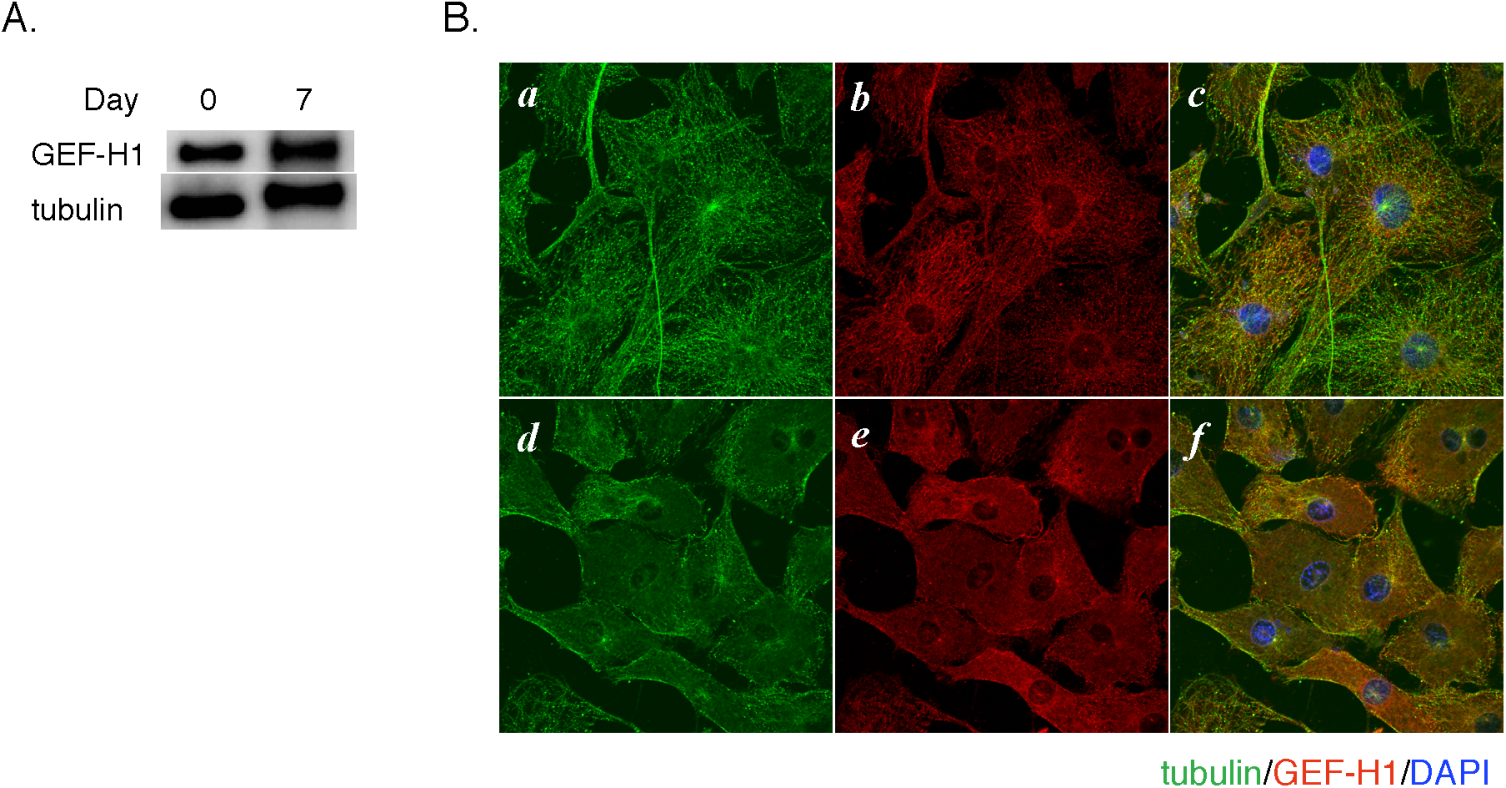
Expression and localization of GEF-H1. A. Cell lysates from 3T3-L1 preadipocytes (Day 0) or differentiated adipocytes (Day 7) were subjected to SDS-PAGE and immunoblotting with anti-GEF-H1 and anti-β-tubulin antibodies. B. 3T3-L1 preadipocytes grown on a cover glass were treated without (*a*-*c*) or with (*d*-*f*) sucralose (20 mM) for 2 hours. After fixation with 100% methanol at -20°C for 2 minutes, cells were immunostained for β-tubulin (green, *a* and *d*) and GEF-H1 (red, *b* and *e*). Cell nuclei were visualized with DAPI (blue) in the merged images (*c* and *f*).

In the next series of experiments, we investigated the relationship between microtubule integrity and Rho activity. To this end, we first examined the expression and localization of GEF-H1 in 3T3-L1 cells. As shown in Fig 3A, GEF-H1 was expressed both in preadipocytes (Day 0) and differentiated adipocytes (Day 7). Immunofluorescence microscopy revealed that GEF-H1 was stained as filamentous signals that were co-localized with the microtubules in preadipocytes (Fig 3Ba–3Bc). Sucralose stimulation induced disruption of the microtubule network, which was associated with diffuse distribution of GEF-H1 in the cytoplasm (Fig 3Bd–3Bf).

**Fig 4:**
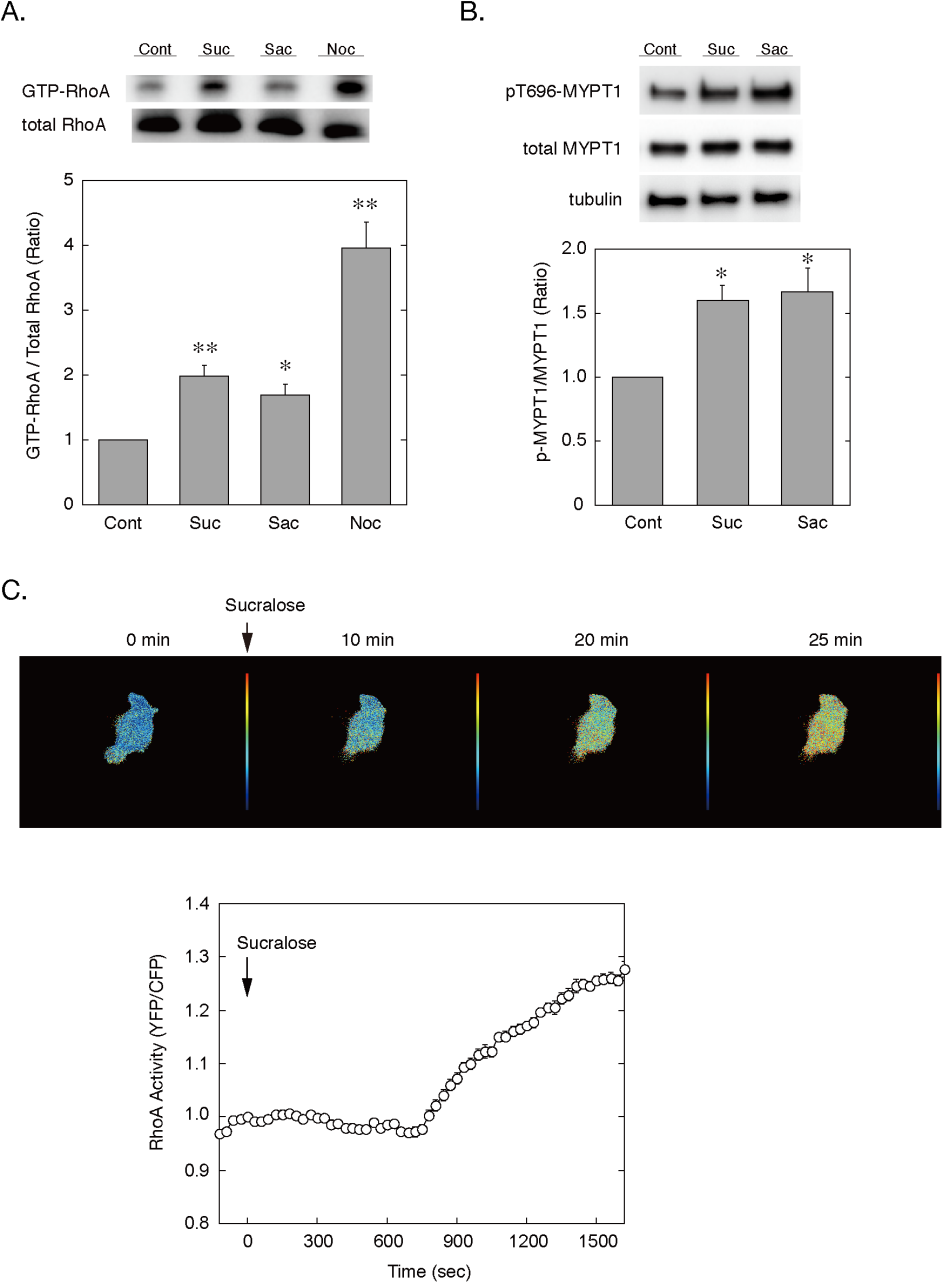
Sweetener activation of the Rho-ROCK pathway. A. 3T3-L1 preadipocytes were stimulated without (Control) or with sucralose (20 mM), saccharin (20 mM) or nocodazole (5 μM) for 2 hours. Cells were lysed and subjected to the pull-down assay of RhoA. Representative immunoblot data for total and GTP-bound RhoA are shown in the upper panel. The intensities of the bands were quantified and the relative RhoA activities are shown as the GTP-bound to total RhoA ratios in the lower panel. Results are shown as the mean ± SEM (n = 4). *, p < 0.05; **, p < 0.005. Con, control; Suc, sucralose; Sac, saccharin; Noc, nocodazole. B. 3T3-L1 preadipocytes were stimulated without (Control) or with sucralose (20 mM) or saccharin (20 mM). The amounts of phospho-MYPT1, MYPT1 and β-tubulin at 4 hours were examined by immunoblotting. Representative immunoblot data (upper panel) and the relative amounts of phospho-MYPT1 normalized with total MYPT1 (lower panel) are shown. Results are shown as the mean ± SEM (n = 4). *, p < 0.05. Con, control; Suc, sucralose; Sac, saccharin. C. 3T3-L1 preadipocytes transfected with Raichu1237X were stimulated with sucralose (20 mM), and dual emission images for CFP and YFP were obtained every 30 seconds. The calculated YFP/CFP emission ratio images at the indicated time points are shown in pseudo-color in the upper panel. The graph shows the change in the emission ratio of YFP/CFP in the cytoplasm. Results are shown as the mean ± SEM (n = 8) from multiple ROIs.

We next measured the Rho activity by pull-down assay using GST-Rhotekin-RBD fusion protein as well as by real-time monitoring using a FRET-based Rho probe, Raichu1237X. Stimulation with sucralose or saccharin of 3T3-L1 preadipocytes increased the amount of GTP-bound RhoA (Fig 4A). These effects may be due to microtubule disassembly, since treatment of cells with nocodazole, a microtubules disrupting reagent, also increased GTP-bound RhoA. Additionally, sucralose and saccharin stimulated phosphorylation of MYPT1, a substrate of Rho-associated kinase (ROCK), suggesting that the sweeteners activated the Rho-ROCK pathway (Fig 4B). Furthermore, the activation of Rho was further supported by the real-time monitoring using Raichu1237X, which revealed that sucralose stimulation progressively activated RhoA after a lag time of ~12 minutes in preadipocytes (Fig 4C).

**Fig 5:**
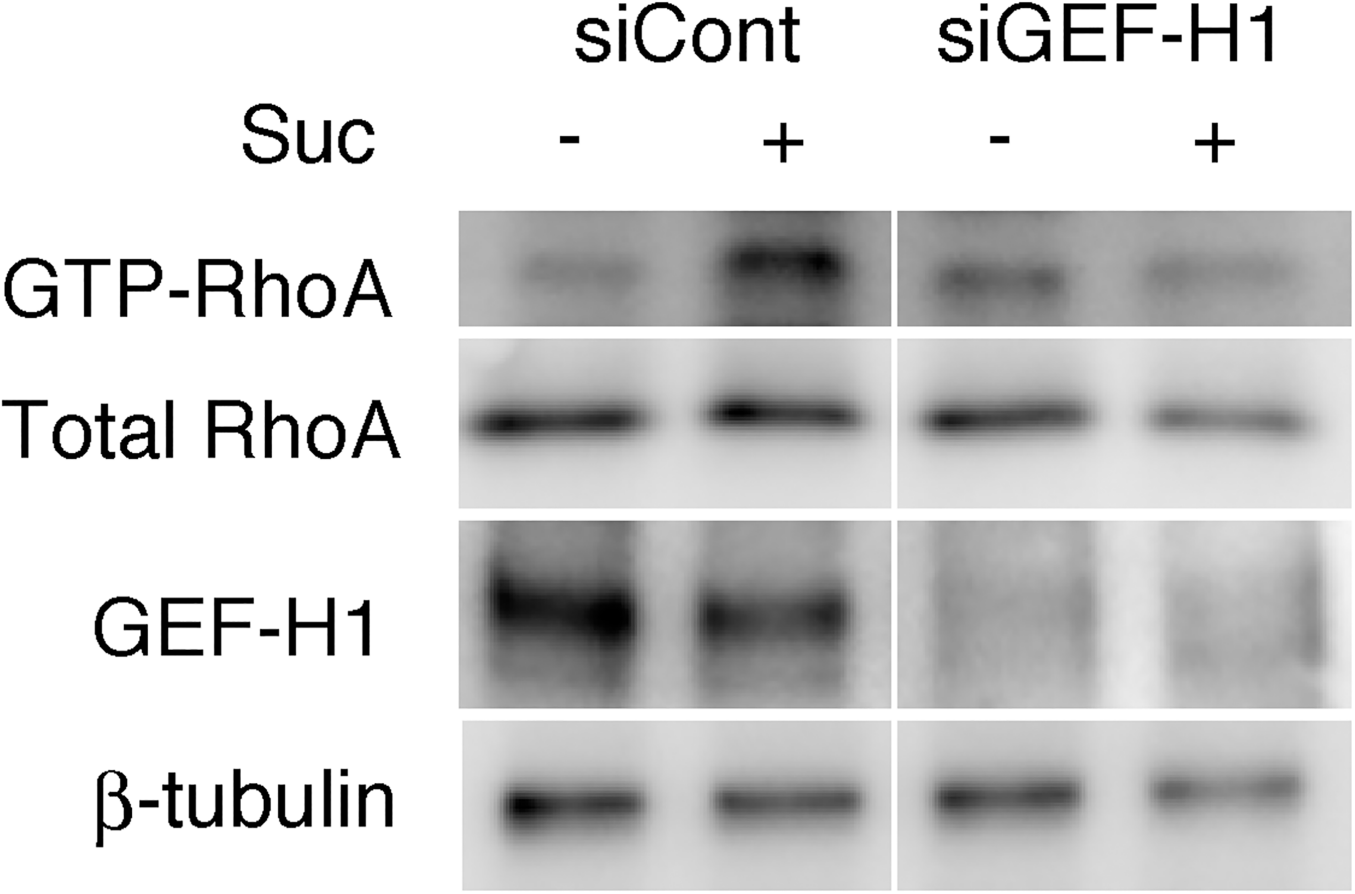
Role of GEF-H1 in sweetener-induced Rho activation. 3T3-L1 cells were transfected with non-targeting (siCont) or GEF-H1-targeting (siGEF-H1) siRNA (5 nmole, each) and cultured for 24 hours before stimulation with sucralose (Suc) for 2 hours. RhoA activity was measured by pull-down assay as described in Fig 4. Immunoblot data illustrate the amount of pull-downed GTP-bound RhoA and the amount of total RhoA, GEF-H1 and β-tubulin in the cell lysates.

We also examined the role of GEF-H1 in the sweetener-induced Rho activation by siRNA-mediated knockdown of GEF-H1. As depicted in Fig 5, transfection of the GEF-H1-targeted siRNA duplexes reduced the amount of GEF-H1 by 75%. Under these conditions, the sucralose-induced activation of RhoA was significantly attenuated, suggesting that GEF-H1 would play a critical role as a link between microtubules disassembly and Rho activation in the signaling cascade downstream of the T1R3 homomeric receptor.

**Fig 6:**
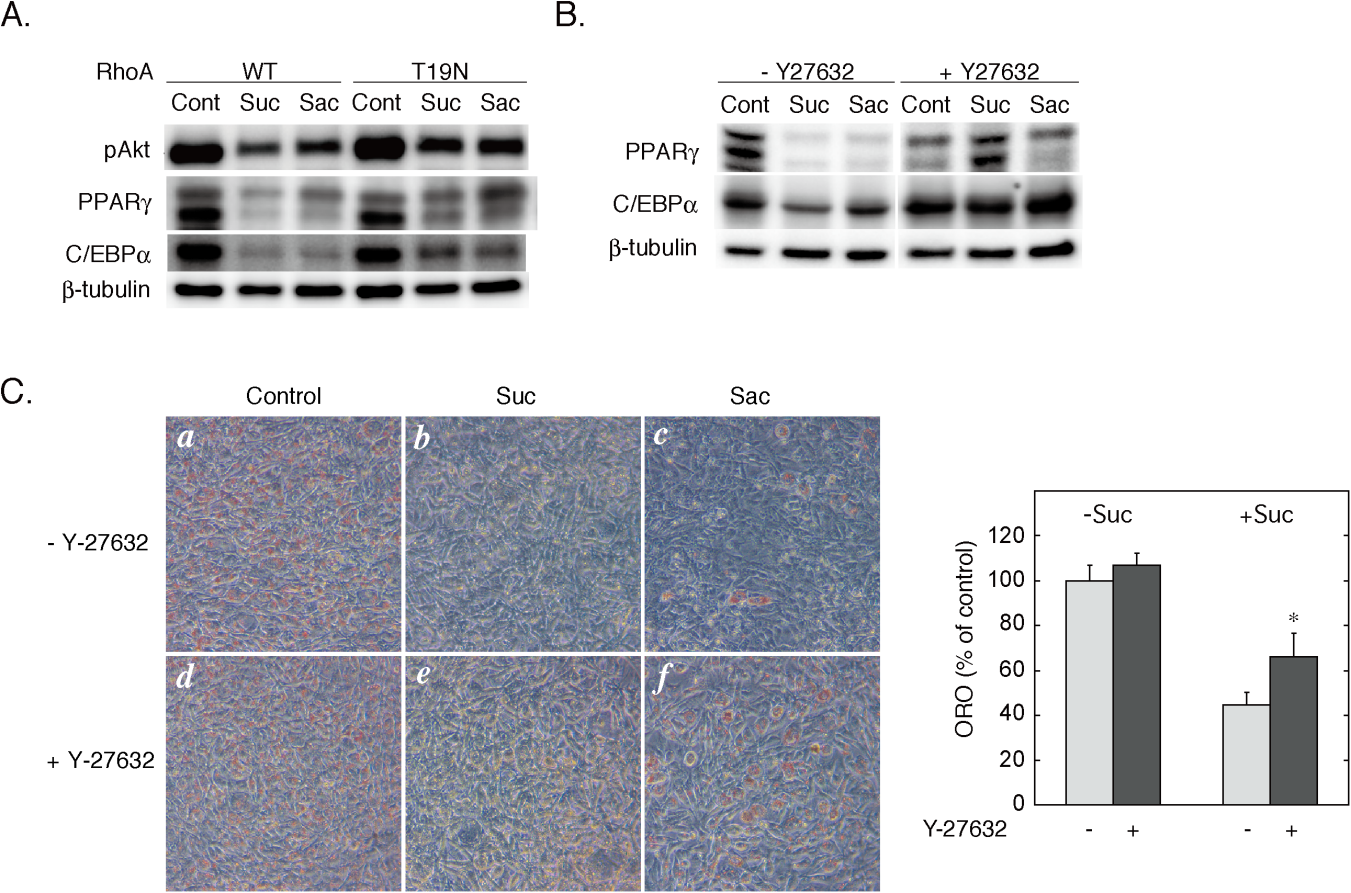
Roles of the Rho-ROCK pathway in sweetener-induced dephosphorylation of Akt and repression of adipogenic transcription factors. A. 3T3-L1 preadipocytes were transfected with the expression plasmids (30 μg each) for wild-type or T19N mutant RhoA by electroporation. Cells were cultured to confluence before the induction of differentiation without (control) or with sucralose (20 mM) or saccharin (20 mM). The amount of phospho-Akt and the expression levels of PPARγ and C/EBPα at 48 hours were measured by immunoblotting. B. 3T3-L1 preadipocytes were differentiated without (control) or with sucralose (20 mM) or saccharin (20 mM) in the absence or the presence of Y-27632 (20 μM). The expression levels of PPARγ and C/EBPα at 48 hours were measured by immunoblotting. C. 3T3-L1 cells were differentiated without (Control) (*a* and *d*) or with sucralose (20 mM) (*b* and *e*) or saccharin (20 mM) (*c* and *f*), in the absence or the presence of Y-27632 (30 μM) during the first 48 hours of differentiation, and were stained with Oil- Red-O at Day 6. Microscopic images of Oil Red-O stained cells were shown in the left panel. The amounts of Dye were quantified as described 'Materials and Methods' and shown in the right panel. Results are shown as the mean ± SEM (n = 3). *, p < 0.05 vs. control (without Y-27632).

In the final set of experiments, we investigated the role of Rho in the anti-adipogenic effect of sweeteners. Previous studies have shown that Rho GTPase is a negative regulator of adipogenesis [8–11]. In 3T3-L1 preadipocytes transfected with wild-type RhoA, sucralose and saccharin attenuated the expression of PPARγ and C/EBPα (Fig 6A) at 48 hours of differentiation, consistent with our previous observation [5]. In contrast, overexpression of the dominant-negative mutant of RhoA (RhoA-T19N) partially blocked the repressive effects of sweeteners on PPARγ and C/EBPα (Fig 6A). Since the PI3K-Akt pathway plays a pivotal role in the adipogenesis by regulating the expression of the adipogenic transcription factors [19–23], we examined whether sweetener stimulation would affect the phosphorylation of Akt. As shown in Fig 6A, sucralose and saccharin induced dephosphorylation of Akt in RhoA-WT-overexpressed cells, which was partially attenuated by overexpression of RhoA-T19N. Additionally, Y-27632, a specific inhibitor of ROCK, restored the repressive effects of sucralose and saccharin on PPARγ and C/EBPα (Fig 6B). Furthermore, we examined the role of the Rho-ROCK pathway in sweetener-inhibition of adipogenesis by Oil-Red-O staining. As shown in Fig 6C, the addition of sucralose or saccharin during the first 48 hours of differentiation significantly inhibited the accumulation of triglyceride at Day 6, which was partially blocked in the presence of Y-27632. These results suggest that Rho plays a critical role in the T1R3 homomeric sweet taste receptor-mediated inhibition of adipogenesis.

## Discussion

In the present study, we investigated the mechanism of sweet taste receptor-mediated inhibition of adipogenesis in 3T3-L1 cells. The results support a model that stimulation of T1R3 homomeric sweet taste receptor activates the Rho GTPase through Gαs-mediated microtubule disassembly and consequent activation of GEF-H1, a microtubule-localized guanine nucleotide exchange factor for Rho. The activated Rho would cause Akt inhibition and repression of the adipogenic transcription factors, PPARγ and C/EBPα. First, immunofluorescence microscopy data showed that sucralose and saccharin treatment caused disassembly of the microtubules, which was mimicked by Gαs activators, such as cholera toxin and isoproterenol, but not with forskolin. Thus, the effects of sweeteners on the microtubules were dependent on the activity of Gαs but not on the increase of cAMP. The involvement of Gαs in the microtubules disassembly was further supported by the inhibition and the mimicry of the effects of sweeteners with the dominant-negative and constitutively active mutants of Gαs, respectively. In addition, a real-time monitoring study revealed a rapid change in the subcellular localization of Gαs-GFP from the plasma membrane to the cytoplasm by stimulation with sucralose. These results are in agreement with our previous observations that the T1R3 homomeric sweet taste receptor in 3T3-L1 cells is coupled to Gs but not to gustducin [5]. Second, the measurement of Rho activity showed that Gαs-mediated disassembly of the microtubules was accompanied by the activation of RhoA, which was mimicked by nocodazole-treatment and attenuated by knockdown of GEF-H1. These data are consistent with the idea that sweetener-induced microtubule disassembly led to Rho activation through the activation of GEF-H1. Additionally, sweetener-induced Rho activation was associated with the activation of ROCK, which was shown by increased phosphorylation of MYPT1, a substrate of ROCK. Finally, our data showed that sweetener-induced Rho activation would be responsible for the inhibition of adipogenesis. Thus, overexpression of the dominant-negative RhoA mutant as well as Y-27632, a specific inhibitor of ROCK, rescued the sweetener-induced repression of the adipogenic transcription factors such as PPARγ and C/EBPα in the early phase of adipogenic differentiation. Although the mechanism of Rho-mediated repression of PPARγ and C/EBPα has not been defined in the present study, it would be a possibility that Rho-dependent inhibition of Akt would be involved in the repression of the adipogenic transcription factors (Fig 6) (see below for further discussion). Importantly, however, the dominant-negative RhoA and Y-27632 only partially rescued sweetener-induced repression of PPARγ and C/EBPα, and Y-27632 only partially blocked the inhibition with sweeteners of triglyceride accumulation at Day 6. Thus, although the Rho-ROCK pathway plays a critical role in the signaling cascade downstream of the homomeric T1R3 receptor, other unproven mechanism(s) may work in the inhibition of adipogenesis.

**Fig 7:**
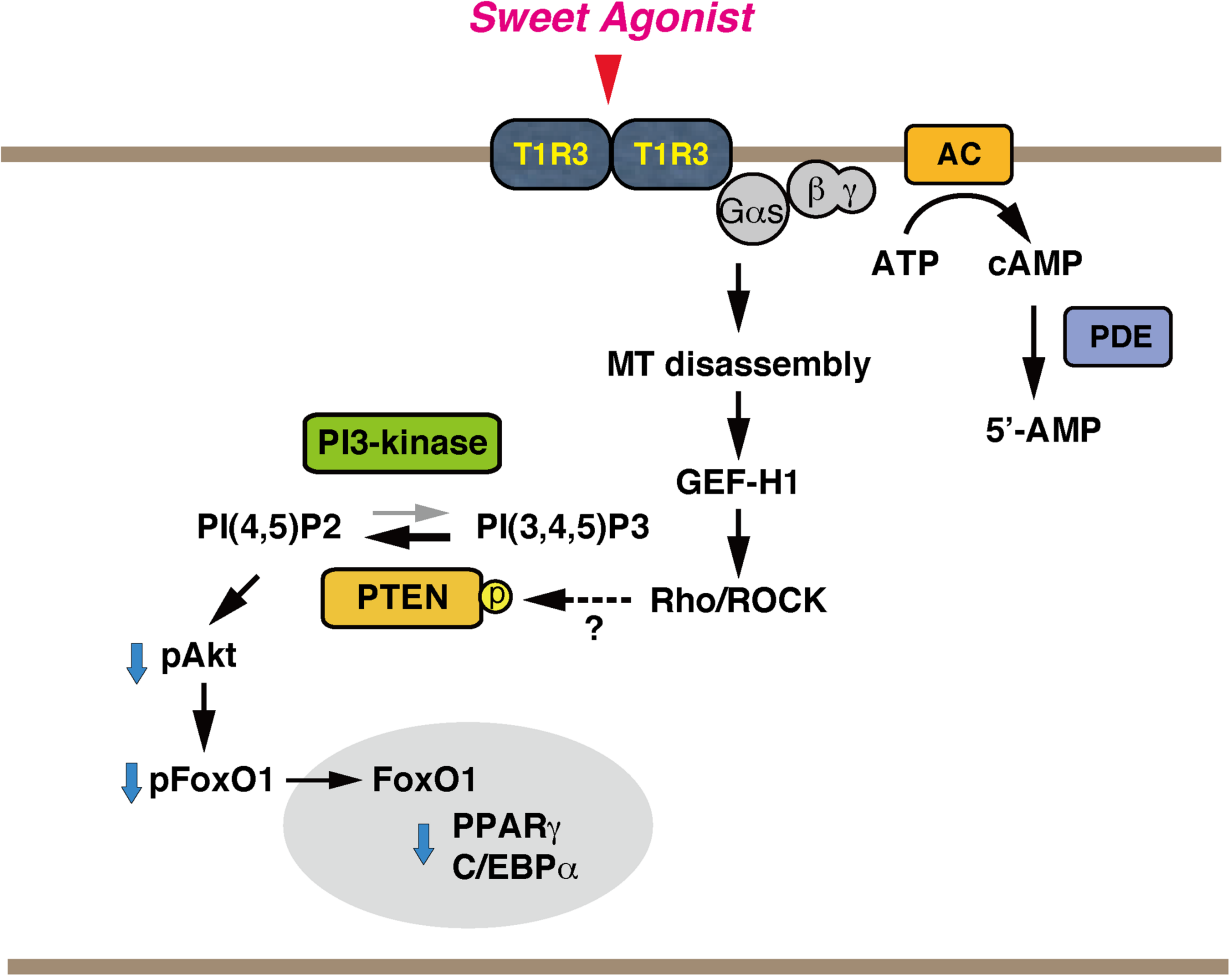
A model for the signaling cascade downstream of the homomericT1R3 sweet taste receptor in 3T3-L1 cells.

These findings revealed a novel signaling cascade downstream of the Gs-coupled T1R3 homomeric sweet taste receptor, which is expressed in preadipocytes as well as in adipocytes [5]. Our present working model for the sweet taste receptor signaling is diagramed in Fig 7. In this model, the signals are relayed from the Gs-coupled T1R3 homomeric receptor to microtubules and to Rho GTPase, which causes a modulation of the PI3K-Akt pathway, thus inhibiting adipogenesis. One of the interesting features of this model is that the anti-adipogenic signal depends on the unique non-canonical role of GTP-bound Gαs that activates the tubulin GTPase and destabilizes microtubules [24] but not on the Gαs-adenylyl cyclase pathway. In this regard, the observation that isoproterenol, a β-adrenergic receptor agonist, also caused microtubule disassembly raised a possibility that long-term stimulation of Gs-coupled receptor may modify the cytoskeleton in certain cell types, which would provide us a new perspective on the interpretation of the data[18].

3T3-L1 preadipocytes express non-canonical T1R3 homomeric sweet taste receptor, which is coupled to Gs. Stimulation of this receptor causes microtubule disassembly in a Gαs-dependent but cAMP-independent manner possibly by stimulation of the tubulin GTPase with the active Gαs. Microtubule disassembly leads to the release of the microtubule-localized RhoGEF, GEF-H1, which activates the Rho-ROCK pathway. The activated Rho would repress adipogenic transcription factors through dephosphorylation of Akt and FoxO1 in the early stages of adipogenesis.

Note that the mechanisms of Rho-mediated dephosphorylation of Akt and repression of PPARγ and C/EBPα have not been fully defined in the present study. One possibility is that Rho may attenuate the insulin signaling cascade. In this regard, Noguchi et al. argued that Rho inhibits adipogenesis by reducing the insulin signaling through ROCK2-mediated phosphorylation of IRS-1 at serine residues [10]. Alternatively, taking into consideration that the PI3K activity is antagonized by the phosphatase and tensin homologue deleted on chromosome 10 (PTEN) and that the stability and activity of PTEN is regulated by post-translational modifications [25], it is also possible that ROCK would activate or stabilize PTEN by phosphorylation [26], antagonizing PI3K-mediated Akt phosphorylation. Inhibition of the PI3K-Akt pathway would lead to dephosphorylation and nuclear translocation of FoxO1, which may be partly responsible for the repression of PPARγ [27–29]. Further study will be needed to clarify this point.

In summary, we report herein a unique signaling cascade downstream of the Gs-coupled T1R3 homomeric sweet taste receptor, which activates the Rho-ROCK pathway and negatively regulates adipogenesis in 3T3-L1 cells. Since this receptor is expressed abundantly in mature adipocytes [5], the receptor-evoked cytoskeletal derangement, Rho activation and Akt inhibition may be relevant to the physiological and pathological functions of adipocytes such as insulin-stimulated glucose uptake and secretion of adipokines. At present, the physiological endogenous ligand(s) for this receptor remains to be defined since its affinity to known physiological sweet compounds in the extracellular milieu such as glucose and sweet amino acids seems very low. Nevertheless, since the Rho-ROCK pathway is activated in obesity and its inhibition ameliorates various metabolic derangements in obesity [30–32], pharmacological inhibition of this receptor may provide a therapeutic strategy for obesity-related disorders.
