# Correction: T1R3 homomeric sweet taste receptor regulates adipogenesis through Gαs-mediated microtubules disassembly and Rho activation in 3T3-L1 cells

**DOI:** 10.1371/journal.pone.0181293

**Published:** 2017-07-07

**Authors:** Yosuke Masubuchi, Yuko Nakagawa, Johan Medina, Masahiro Nagasawa, Itaru Kojima, Mark M. Rasenick, Takeshi Inagaki, Hiroshi Shibata

A reference is omitted from the second sentence of the second paragraph in the Introduction.

The sentence should read: Thus, in contrast to our observations, Simon et al. reported that saccharin and acesulfame potassium stimulated adipogenesis in mouse mesenchymal stem cells and human preadipocytes although the effects were independent of T1R2 and T1R3 (Simon et al., 2013).

The reference is: Simon BR, Parlee SD, Learman BS, Mori H, Scheller EL, Cawthorn WP, et al. Artificial sweeteners stimulate adipogenesis and suppress lipolysis independently of sweet taste receptors. J Biol Chem. 2013;288(45):32475–89. doi: 10.1074/jbc.M113.514034. PubMed PMID: 24068707; PubMed Central PMCID: PMCPMC3820882.

A reference is omitted from the third sentence of the second paragraph in the Introduction.

The sentence should read: The same group has shown that both T1R2 and T1R3 knockout mice have reduced adiposity and smaller adipocytes on a Western Diet (Simon et al., 2014).

The reference is: Simon BR, Learman BS, Parlee SD, Scheller EL, Mori H, Cawthorn WP, et al. Sweet taste receptor deficient mice have decreased adiposity and increased bone mass. PLoS One. 2014;9(1):e86454. doi: 10.1371/journal.pone.0086454. PubMed PMID: 24466105; PubMed Central PMCID: PMCPMC3899259.

A reference is omitted from the penultimate sentence of the second paragraph in the Introduction.

The sentence should read: Additionally, those T1R3 deficient mice were with mild glucose intolerance and no changes in insulin sensitivity, while Murovets et al. reported that T1R3 knockout mice on a standard diet showed substantially reduced glucose tolerance and insulin sensitivity (Murovets, Bachmanov, & Zolotarev, 2015).

The reference is: Murovets VO, Bachmanov AA, Zolotarev VA. Impaired Glucose Metabolism in Mice Lacking the Tas1r3 Taste Receptor Gene. PLoS One. 2015;10(6):e0130997. doi: 10.1371/journal.pone.0130997. PubMed PMID: 26107521; PubMed Central PMCID: PMCPMC4479554.
